# Observation of site-controlled localized charged excitons in CrI_3_/WSe_2_ heterostructures

**DOI:** 10.1038/s41467-020-19262-2

**Published:** 2020-10-30

**Authors:** Arunabh Mukherjee, Kamran Shayan, Lizhong Li, Jie Shan, Kin Fai Mak, A. Nick Vamivakas

**Affiliations:** 1 The Institute of Optics, University of Rochester, Rochester, NY USA; 2 School of Applied and Engineering Physics, Cornell University, Ithaca, NY USA; 3 Kavli Institute at Cornell for Nanoscale Science, Ithaca, NY USA; 4 Laboratory of Atomic and Solid State Physics, Cornell University, Ithaca, NY USA; 5 Materials Science, University of Rochester, Rochester, NY USA; 6 Department of Physics and Astronomy, University of Rochester, Rochester, NY USA; 7 Center for Coherence and Quantum Optics, University of Rochester, Rochester, NY USA

**Keywords:** Nanoscience and technology, Optics and photonics

## Abstract

Isolated spins are the focus of intense scientific exploration due to their potential role as qubits for quantum information science. Optical access to single spins, demonstrated in III-V semiconducting quantum dots, has fueled research aimed at realizing quantum networks. More recently, quantum emitters in atomically thin materials such as tungsten diselenide have been demonstrated to host optically addressable single spins by means of electrostatic doping the localized excitons. Electrostatic doping is not the only route to charging localized quantum emitters and another path forward is through band structure engineering using van der Waals heterojunctions. Critical to this second approach is to interface tungsten diselenide with other van der Waals materials with relative band-alignments conducive to the phenomenon of charge transfer. In this work we show that the Type-II band-alignment between tungsten diselenide and chromium triiodide can be exploited to excite localized charged excitons in tungsten diselenide. Leveraging spin-dependent charge transfer in the device, we demonstrate spin selectivity in the preparation of the spin-valley state of localized single holes. Combined with the use of strain-inducing nanopillars to coordinate the spatial location of tungsten diselenide quantum emitters, we uncover the possibility of realizing large-scale deterministic arrays of optically addressable spin-valley holes in a solid state platform.

## Abstract (web summary)

Quantum emitters in atomically thin materials host optically addressable single spins. Here, the authors demonstrate spin selectivity in the preparation of the spin-valley state of site-controlled localized single holes in CrI3/WSe2 heterostructures.

## Introduction

Photonic integrated circuits are advancing the state-of-the-art in quantum and classical information processing due to desirable properties like high operating speeds and low energy consumption^1^. In this context, atomically thin semiconducting transition metal dichalcogenides (TMDs) have emerged as a unique platform that offers a material interface to photon polarization via the coupled spin–valley degree of freedom of its charge carriers^2–5^. Consequently, TMD-based optically driven valleytronic devices^6^ have gained interest as potential candidates for beyond complementary metal-oxide-semiconductor (CMOS) technology. These devices operate by coupling the different circular polarizations of light with degenerate inequivalent local energy extrema (referred to as valleys^3–5^) in the electronic band structure—valley specific addressing is accomplished via the handedness of light polarization^3–8^.

Due to reduced dielectric screening in the two-dimensional (2D) plane, excitonic species strongly dominate the optical response of atomically thin TMDs^9,10^. 2D excitons, like the neutral (X^0^) and charged excitons (X^±^, also known as trions), have been shown to inherit the valley character from the single-particle electronic band structure^3–8,11^. Interestingly, the valley physics holds even for the case of localized neutral excitons^12–15^ and trions^16–18^, provided the confinement length scale is larger than the Bohr radius of the excitons(~1 nm). A localized positive trion, for example, can be described as a bound state of three charges, an electron and a hole in one valley and another hole in the opposite valley. Such a localized trion radiatively relaxes to a ground state of one spin–valley hole—the detection of a circularly polarized photon is a projective measurement of the hole into one of the two possible spin–valley states. It is predicted that this hole will preserve its spin orientation for at least a few ns^17^ which may be sufficient for performing several quantum gate operations, provided that appropriate steps like pulsed optical excitation and post-selection to disregard trion re-trapping events are performed.

Although generating quantum confined trions in tungsten diselenide (WSe_2_) has been previously realized by electrostatic doping^16–18^ in a van der Waals heterostructure (HS), the lack of ability to position the quantum emitter and the use of significant bias voltage for electrostatic doping limits the scalability and economical fabrication of optically driven valleytronic devices. Furthermore, electrostatic doping is not spin-selective, thus preventing any spin selectivity of the trions unless polarization selection schemes in the excitation and collection optics are used. Thus, spin-oriented quantum confined trions, excited by means of band-structure engineering, provides a pathway to circumvent the previous. Recently, it has been shown that 2D positive trions in a type-II HS can be formed by coupling chromium triiodide (CrI_3_) thin films to a monolayer WSe_2_^19^. Spin-polarized charge transfer between CrI_3_ and WSe_2_ results in a finite intensity contrast between the opposite circularly polarized positive trions even at no applied magnetic fields. The previous points to the possibility of forming site-controlled localized charged excitons with spin selectivity in WSe_2_/CrI_3_ if placed onto patterned nanopillar arrays^20,21^.

In this work, we use band-structure engineering to create localized X^+^ in monolayer WSe_2_ which is interfaced with bilayer CrI_3_. The type-II band alignment between the two materials promotes p-doping of WSe_2_ via charge transfer. We first show the existence of charge transfer using photoluminescence (PL) from the 2D positive trion. At locations of strain-inducing nanopillars, we observe quantum emitters, positive trions, that do not exhibit fine-structure splitting (FSS) due to quenched electron–hole exchange interactions—this is the hallmark of 0D trions^17^. We confirm the trionic character by using polarization-resolved magneto-PL experiments. We also demonstrate finite spin selectivity in the 0D X^+^ at no applied magnetic fields due to the spin-dependent charge transfer in the HS. Our work thus demonstrates site-controlled localized trions with spin selectivity in atomically thin materials.

## Results

### Optical response of the device on and off nanopillars

**Fig. 1:**
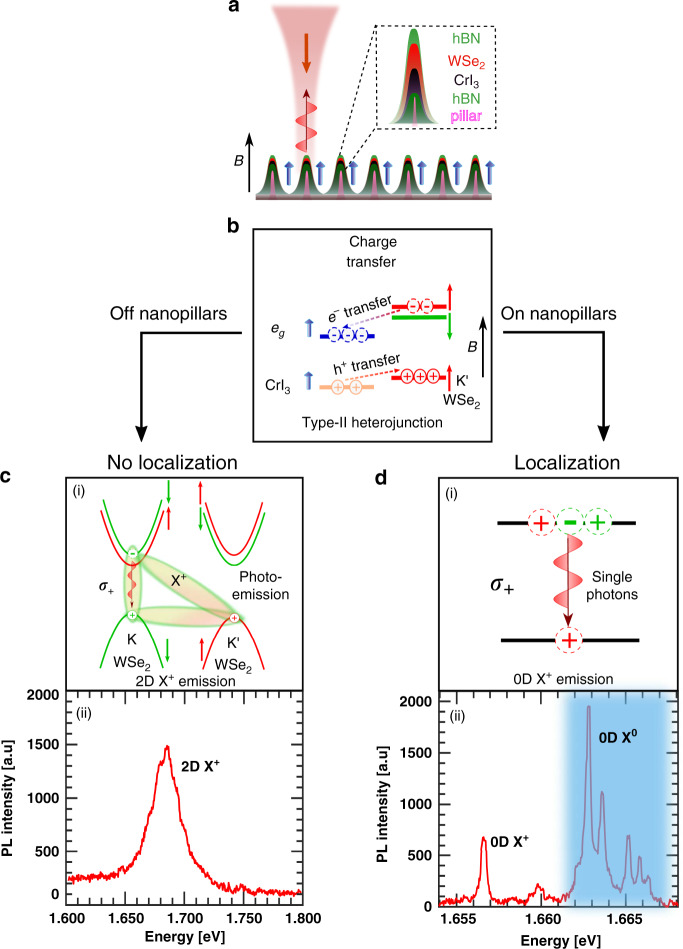
CrI_3_/WSe_2_ heterostructure stamped onto nanopillar arrays. **a** A schematic illustration of the device structure showing the ordering of the atomically thin layers in the device. Boxed inset zooms into the region of one nanopillar. **b** Charge-hopping due to Type-II heterojunction results in highly p-doped WSe_2_. Spins of the conduction and valence bands of WSe_2_ (CrI_3_) are shown with red and green (blue) arrows. **c** Photo-emission process of 2D X^+^ with an extra hole in the K' valley (top); A typical PL spectrum of the heterostructure off the nanopillars showing the prominence of the 2D X^+^ emission due to hole doping at no applied magnetic field (bottom). **d** Localization emission process for a 0D X^+^ leading to a single hole in the K' valley(top); PL spectrum at zero magnetic field on the nanopillars showing multiple 0D X^+^ and 0D X^0^ emission lines (bottom).

A schematic of our van der Waal HS device is shown in Fig. 1a. An optical micrograph of the device is shown in Supplementary Fig. 1. The HS is placed on a nanopillar array inducing strain on the HS at specific locations. The nanopillar array promotes localization of single excitons which is of central importance in this work. A bilayer CrI_3_ is exfoliated and layered with monolayer WSe_2_ in an N_2_ environment and encapsulated with hBN (see “Methods”). A consequence of the type-II band alignment between WSe_2_ and CrI_3_ is charge-hopping of charge carriers that results in hole doping of WSe_2_^19^. Figure 1b demonstrates how the type-II band alignment between WSe_2_ and CrI_3_ can promote hole doping of WSe_2_. This is manifested in the dominance of positively charged excitonic species PL. We show typical PL spectra on and off nanopillars in Fig. 1c, d in the HS region. The broad spectrum shown in Fig. 1c is consistent with previous observations^19,22^ and can be attributed to delocalized X^+^ species. In contrast to this broad spectrum, in the vicinity of the nanopillars, we observe multiple narrow linewidth emission lines. The strain gradients induced by the nanopillars lead to such sharp emission lines with linewidths of 200–500 μeV. Some of the emission lines occur in pairs (doublets) while there also are emissions with only one peak (singlets) at no applied magnetic field. The doublets are due to localized neutral excitons (0D X^0^). The FSS occurs due to anisotropic electron and hole exchange interactions in an asymmetric confinement potential. In contrast, the positively charged 0D X^+^ in WSe_2_ exhibits a singlet hole-spin configuration that quenches the electron–hole exchange interactions. A tell-tale signature of localized charged excitons is the absence of FSS at zero magnetic field^17^.

To explain the origin of the charge-hopping between WSe_2_ and CrI_3_, we consider the spatial band-diagram in Fig. 1b. The WSe_2_ band structure consists of valleys at the six corners of the first Brillouin Zone. The valleys located at the opposite edges (labeled as K and K′) can be accessed using opposite circularly polarized photons (*σ*_±_). This phenomenon, known as spin–valley locking, stems from spin–orbit interactions that renders both the conduction and valence bands of each valley spin-polarized. The red and green levels shown are spin up and down, respectively. We also show the conduction and valence band levels of CrI_3_. The lowest unoccupied conduction band consists of e_*g*_ levels^23,24^ pointing in the direction of the applied magnetic field^19^. As a result these empty spin-polarized states e_*g*_ orbitals (shown in blue) can accept the spin-polarized electrons from one of the K or K′ valleys. For the K′ valley of WSe_2_, electrons prefer to move to CrI_3_ whereas the holes move in the opposite way, thereby p-doping WSe_2_. Consequently, at high excitation powers, the PL spectrum is dominated by delocalized positively charged exciton species X^+^ which is composed of two holes located in opposite valleys and one electron in one of those valleys. Furthermore, this type of charge distribution is valley sensitive and is weaker for the K valley in WSe_2_ when the applied magnetic field pointing in the +*z* direction. This leads to brighter PL from X^+^ involving the electrons located in the K valley. The photo-emission process in the energy–momentum space is depicted in Fig. 1c(i). The recombination of the e–h pair releases a circularly polarized photon and the extra hole is left behind the K′ valley. The PL spectrum of the delocalized X^+^ from a region between two nanopillars in the HS is shown in Fig. 1c(ii).

We now employ the charge-hopping phenomenon to explain the observation of charged single localized excitons. It has been shown that strain, induced by means of nanopillars^20,21^, or randomly occurring nano-bubbles^25^, facilitate the localization of neutral excitons (0D X^0^). Upon significant p-doping, the 0D X^0^ undergo a hole-capture process to result in 0D X^+^. Unlike previous reports^17,18^, our device architecture circumvents the need for electrical gating. The excited state of 0D X^+^, akin to the delocalized 2D X^+^, consists of two holes and a single electron which upon radiative relaxation leaves behind a single localized hole. The spin of this hole is determined by the circular polarization of the emitted photon. The process for a spin-up extra hole has been shown in Fig. 1d(i) and an exemplary PL spectra on a nanopillar site shown in Fig. 1d(ii).

### Spin-dependent charge transfer in the CrI_3_/WSe_2_ HS

**Fig. 2:**
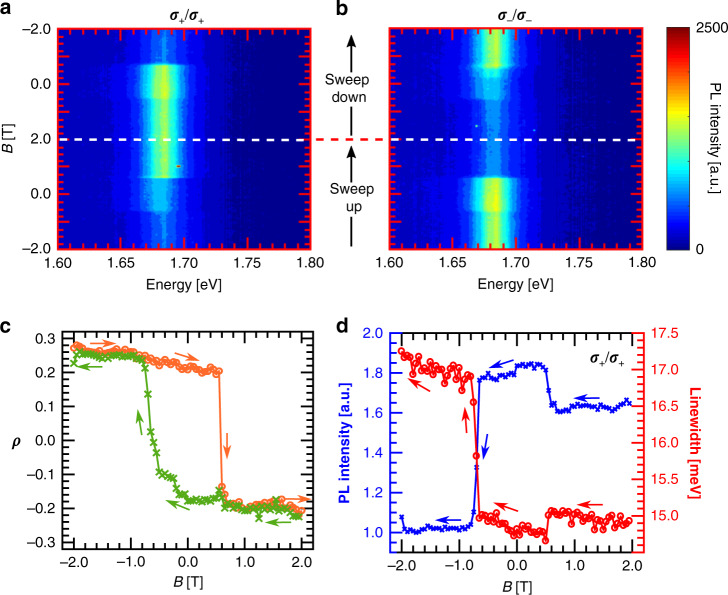
Charge transfer in CrI_3_WSe_2_ heterostructure. **a**, **b** Magneto-PL intensity of the 2D X^+^ for right (*σ*_−_) and left (*σ*_+_) circular excitation and collection. The charge transfer is valley selective causing the K and K' valleys to have unequal PL intensity, depending on the spin CrI_3_ interfacial layer. The magnetic field is sweeped in a loop between  ±2 T. The white dashed line represents the field at which the field sweep direction is inverted. **c** The interfacial CrI_3_ layer switches spin hysteretically, which is reflected in the PL polarization *ρ*. The orange (green) curves show *ρ* for downward (upward) magnetic field sweeps. **d** The linewidth (red) and intensity (blue) of X^+^ of the K valley changes with applied field. There is a correlated increase of linewidth and decrease of intensity.

To experimentally prove the existence of spin-dependent charge transfer between CrI_3_ and WSe_2_, we performed circular polarization-resolved magneto-PL measurements as described in ref. ^19^. We illuminated the sample with a circularly polarized laser that has a power of 50 μW and measured the co-polarized PL from the delocalized positively charged trions (2D X^+^) while sweeping an external magnetic field. The results are shown in Fig. 2a, b. The presence of an extra non-radiative channel reduces the PL intensity from that valley of WSe_2_, which aligns with the spin of CrI_3_. It has been shown in ref. ^19^ that this effect is governed by spin-dependent charge transfer between the interfacial (top) layer of the CrI_3_ and WSe_2_. For quantitative analysis, we measure the PL polarization *ρ* using

1 $$\rho (B)=\frac{{I}_{\sigma -/\sigma -}-{I}_{\sigma +/\sigma +}}{{I}_{\sigma -/\sigma -}+{I}_{\sigma +/\sigma +}},$$

which is shown in Fig. 2c. Here *I*_*σ*+/*σ*+_(*I*_*σ*−/*σ*−_) corresponds to PL intensity for *σ*_+_(*σ*_−_) excitation and collection. When the spin of top CrI_3_ is in the −*z*(+*z*) direction, the PL from the K′(K) valley is brighter. This leads to a positive (negative) *ρ* at high magnetic fields in the −*z*(+*z*) direction. The top layer CrI_3_ undergoes a spin-flip transition at  ±0.6 T, which is evident from the sudden jump in *ρ* at those fields. This also matches well with a typical magnetic field-dependent magnetic circular dichroism trace of bilayer CrI_3_ (shown in Supplementary Fig. 2), which also responds to the spin-flip transitions of the CrI_3_ layers. Finally, due to the increased non-radiative decay rate, the linewidth of the 2D X^+^ of one valley should increase in correlation with a decrease in intensity. We demonstrate this in Fig. 2d with PL from the K valley during the downward magnet sweep. We see that at fields where the linewidth drops, the PL intensity increases and vice versa. This unambiguously proves the existence charge transfer between the two materials.

### Observation of site-controlled localized X^+^

**Fig. 3:**
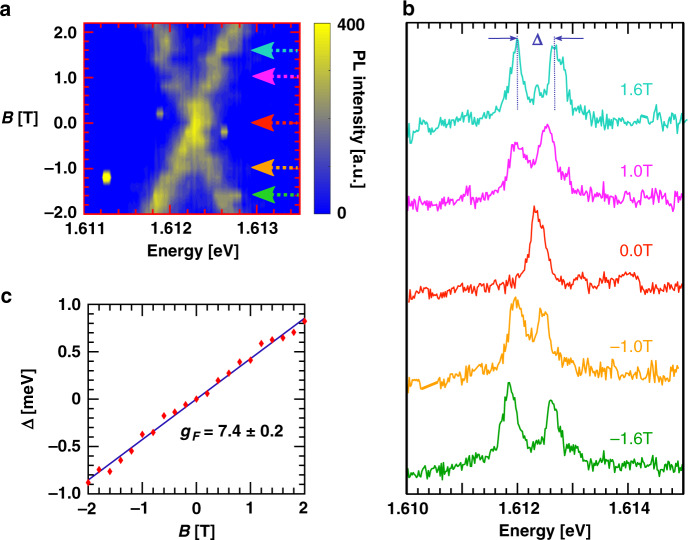
Magneto-PL of 0D X^+^ for linear excitation. The detection does not project into specific polarization states. **a** Colored contour plot of magnetic field-dependent spectrum of 0D X^+^. **b** Spectral linecuts for select fields, marked in **a**, are shown. **c** Splitting between the two peaks shows a linear increase with field without any measurable zero-field splitting.

Having shown the existence of charge transfer, we now exploit it to increase the propensity of observing hole-charged single excitons at strained locations near the nanopillars. We measured the PL spectrum at low excitation laser power (500 nW) of linear polarization at 5 nanopillars and found 15 sharp emission lines without resolvable FSS at zero magnetic field. These nanopillars are identified in an optical micrograph in Supplementary Fig. 1. PL spectra at the corresponding locations are presented in Supplementary Fig. 3. To confirm that these emissions arise from hole-charged excitons, we performed polarization unresolved PL measurements under linear excitation. An exemplary magnetic field-dependent PL spectra is shown Fig. 3a. The characteristic “X” shaped dispersion is observed^17^. Spectral linecuts at select fields are shown in Fig. 3b. The FSS(Δ) is a linear function of applied field: Δ = *g*_*F*_*μ*_B_*B*, where *g*_*F*_ is the Lande’ *g*-factor of 0D X^+^ and *μ*_B_ is the Bohr magneton. For the 0D X^+^ emitter shown here, we found a *g*_*F*_ = 7.4 ± 0.2, consistent with previous reports^18^. On the contrary, the FSS of localized 0D X^0^ shows a starkly different behavior, with a finite zero-field splitting and a quadratic increase at low fields (Supplementary Fig. 4). Furthermore, to rule out the possibility of charged biexcitons we performed power-dependent intensity measurements to investigate the linear response of the PL magnitude to the pump power. Supplementary Figure 5 presents the PL integrated intensity of 0D X^+^, as a function of the excitation power. We observe the power-dependent PL magnitude increases monotonically as *P*^*α*^, where *α* is a fit parameter. From the fit, the value of *α* is 1.02 ± 0.2. Given that charged biexcitonic intensity increase is more quadratic^26^, we can rule out its presence.

### Polarization-resolved magneto-PL of 0D X^+^

**Fig. 4:**
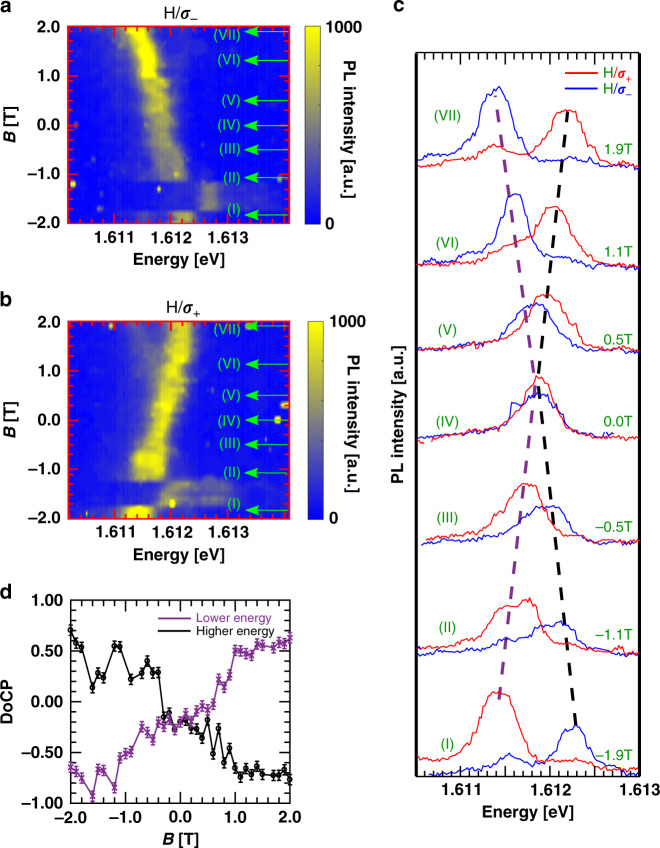
Polarization-resolved magneto-PL for 0D X^+^. Polarization-resolved magneto-PL for linear excitation and **a** *σ*_−_ and **b** *σ*_+_ collection. A linear change in energy of the fine-structure levels confirms positively charged excitonic nature of the localized emission. The purple and black dashed lines follow the lower and higher energy modes respectively. **c** Spectral linecuts at select applied fields as indicated in **a**, **b.** **d** The DoCP of the lower (purple) and higher (black) energy modes extracted from **a** and **b**. The circular polarization purity increases with the separation between the modes.

The fine structure arises from opposite valley-polarized X^+^ modes whose degeneracy is lifted with magnetic field. To confirm this, we performed polarization-resolved magneto-optical measurements for a linearly (circularly) polarized excitation (collection) as a function of applied magnetic fields. Figure 4a, b shows an exemplary polarization-resolved spectral map of 0D X^+^. We find that while the *σ*_+_ shifts downward (upward) in energy at positive (negative) magnetic field, the *σ*_−_ shows the opposite behavior by showing shifts to higher (lower) energy at positive (negative) magnetic field. The sudden energy jump observed at −1.5 T is due to spectral jittering, common in these kind of devices^17^. Figure 4c shows the corresponding polarization-resolved spectral linecuts of the 0D X^+^ at select magnetic fields, recorded with spectral resolution of 20 μeV. At zero field the spectrum clearly shows a single peak with unresolvable FSS. With increasing applied magnetic field, the single peak converts into a doublet and the two components split further apart. We analyzed the degree of circular polarization (DoCP) of each of the modes to study the valley polarization of 0D X^+^. The extracted DoCP values of both higher energy and lower energy peaks are plotted in Fig. 4d at different magnetic fields. The DoCP of the *σ*_+_ and *σ*_−_ are extracted using

2 $${\mathrm{{DoCP}}}=\frac{{I}_{H/\sigma -}-{I}_{H/\sigma +}}{{I}_{H/\sigma -}+{I}_{H/\sigma +}},$$

where *I*_*H*/*σ*+(−)_ is the intensity of the *σ*+ (*σ*−) polarized emissions for linearly polarized excitation. The DoCP values were extracted for the lower and higher energy peaks by integrating the intensity centered at each peak (depicted by purple and black dashed arrows in Fig. 4c) for both the polarized collection spectrum and use the abovementioned equation. We find that with increasing *B* fields, the valley degeneracy is lifted due to Zeeman splitting, and the 0D X^+^ emission becomes increasingly circularly polarized. The energy ordering between the two circularly polarized modes inverts with the field direction.

### PL polarization (*ρ*) of 0D X^+^

**Fig. 5:**
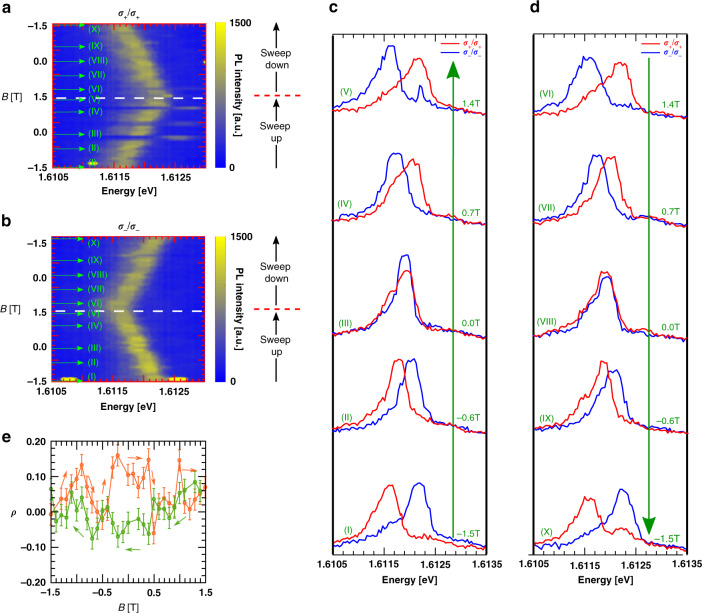
Co-circular polarized magneto-PL of 0D X^+^ spectra for a *σ*_**+**_ and b *σ*_**−**_ collection and excitation. The applied magnetic field is sweeped in a loop between  ±1.5 T. The white dashed lines represent the field at which the sweep direction is inverted. **c**, **d** Spectral linecuts at select applied fields as indicated in **a** and **b**. **e** The PL polarization *ρ* of 0D X^+^ extracted from **a** and **b** using Eq. (2). The orange and green curves represent *ρ* for upward and downward field sweeps.

Finally to investigate the role of the proximal CrI_3_ layer magnetization, akin to the 2D X^+^, we extract the PL polarization (*ρ*) for the 0D X^+^. This is demonstrated in Fig. 5. In contrast to the DoCP measurements where linearly polarized excitation was used, here we recorded PL with co-polarized *circular* excitation and collection as a function of increasing and decreasing magnetic field sweeps. The magneto-optical spectra are shown in Fig. 5a, b. Spectral linecuts at some select fields are shown in Fig. 5c, d. The extracted *ρ* has been shown in Fig. 5e. At zero field, we observe a PL polarization difference of close to 15% between the upward and downward sweeps along with a hysteritic feature the region between ±0.6 T similar to the 2D X^+^ case. The finite intensity contrast in the *σ*_+_ and *σ*_−_ modes at zero field is a result of spin-polarized charge transfer from the CrI3 which prefers positive trion of one polarization over its time reversal pair. This effect is less pronounced for the 0D X^+^ than the 2D 0 X^+^. A plausible explanation can be the uncontrolled contact between the CrI3 and WSe2 near the pillar tips which can lead to a larger separation between the two layers compared to flat regions in between and away from the pillars.

## Discussion

In this work, we have demonstrated the optical excitation of site-controlled 0D X^+^ in WSe_2_ using band-structure engineering. The type-II band alignment between WSe_2_ and CrI_3_ promotes p-doping of WSe_2_ via charge transfer. We first proved the existence of charge transfer using the PL from delocalized positive trions. At locations of strain-inducing nanopillars, we detected localized emission devoid of FSS at zero applied magnetic field. Quenched electron–hole exchange interactions in 0D X^+^ are responsible for such emission spectrum. We confirmed their presence using polarization-resolved and unresolved magneto-PL experiments. We also demonstrated spin selectivity in the 0D X^+^ at zero applied fields, which is a consequence of spin-polarized charge transfer between CrI_3_ and WSe_2_. We envision that our work will fuel further experimental exploration on engineering nano-patterned substrates which maintain the site-control we demonstrated while enhancing the interaction between 0D X^+^ and CrI_3_. A strong magnetically coupled 0D X^+^ can also serve as an optical probe for nano-scale deformation induced change in magnetic order in CrI_3_^27,28^.

## Methods

### Sample fabrication

We fabricated gold nanopillars using e-beam lithography and physical vapor deposition on a p-type Si substrate with 300 nm SiO_2_ passivation. The pillars were 120 nm in height and had a circular cross section of diameter 300 nm. The HS assembly was done using a layer-by-layer dry transfer technique. Atomically thin flakes were first exfoliated from bulk crystals (from HQ Graphene) onto Si substrates with a 300-nm oxide layer. Flakes of monolayer WSe_2_ and bilayer CrI_3_ were identified by their optical contrast. Two thin hBN flakes were chosen to encapsulate the WSe_2_/CrI_3_ HS. Their thickness was less than 3 nm to ensure good contact of the HS to the substrate with nanopillars. The identified flakes were picked up one-by-one in the order of hBN, WSe_2_, CrI_3_, and hBN using a stamp that consists of a polycarbonate thin film attached to a polydimethylsiloxane film on a glass slide. The complete stack was pressed and transferred onto the prepatterned nanopillar substrate by melting the polycarbonate thin film at 180 ^∘^C. The polycarbonate residue on the device was removed with chloroform and isopropyl alcohol before measurements. The fabrication process involving CrI_3_ was performed inside a nitrogen-filled glovebox to avoid oxygen and moisture induced degradation.

### PL measurements

All optical measurements are performed using a home-built confocal microscope. The sample is excited using a 633 nm laser focused onto a diffraction spot size 500 nm using a 0.82 NA objective lens in a closed-cycle cryostat (AttoDry 1000) at 4K equipped with a super-conducting magnet. The PL emitted is collected by the same objective and coupled into a single mode fiber. Polarization selection is carried out using linear polarizers and achromatic quarter wave plates (Thorlabs AQWP10M-580) in appropriate order and relative orientations. The light from the collection fiber is analyzed using a Princeton Instruments spectrometer (Acton SP-2750i) with a resolution limit of 20 μeV.

## Supplementary information

Supplementary Information

## Data Availability

The datasets generated during and/or analyzed during the current study are available from the corresponding author on reasonable request.
