# Supplementary material for: Observation of site-controlled localized charged excitons in CrI3/WSe2 heterostructures
Source: Nat Commun. 2020 Oct 30;11:5502. doi: 10.1038/s41467-020-19262-2 (PMC7599320; doi:10.1038/s41467-020-19262-2)
Supplement: Supplementary file 1 — Supplementary Information [file 41467_2020_19262_MOESM1_ESM.pdf]

Supplementary Information for Observation of site-controlled localized charged excitons in CrI_3/WSe_2 heterostructures

Arunabh Mukherjee[1], Kamran Shayan[1], Lizhong Li[2,3], Jie Shan[2,3,4], Kin Fai Mak[2,3,4] & A. Nick Vamivakas[1,5,6,7,*]

[1] *The Institute of Optics,University of Rochester, Rochester, New York 14627, USA.*

[2] *School of Applied and Engineering Physics, Cornell University, Ithaca, New York 14853, USA.*

[3] *Kavli Institute at Cornell for Nanoscale Science, Ithaca, New York 14853, USA.*

[4] *Laboratory of Atomic and Solid State Physics, Cornell University, Ithaca, NY, USA*

[5] *Materials Science, University of Rochester, Rochester, NY, USA*

[6] *Department of Physics and Astronomy, University of Rochester, Rochester, NY, USA*

[7] *Center for Coherence and Quantum Optics, University of Rochester, Rochester, NY, USA*

**E-mail: nick.vamivakas@rochester.edu*

1 Optical micrograph of the device

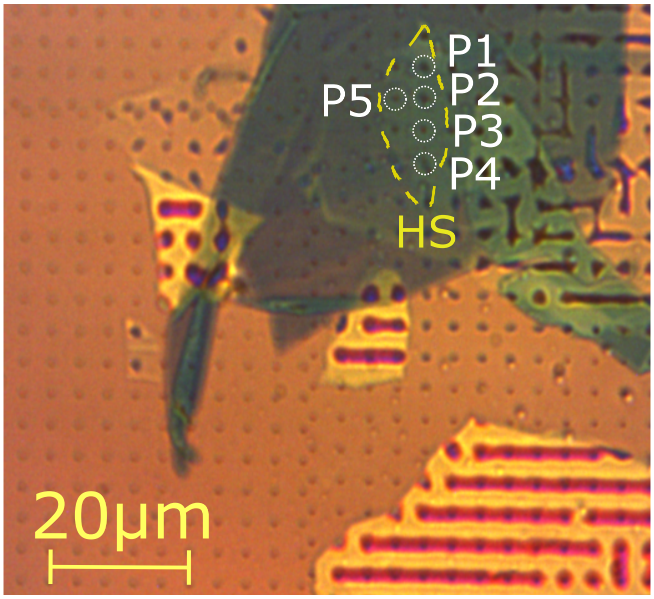

Supplementary Figure 1: Optical image of the van der Waals heterostructure device

Here we show an optical microscope image of the van der Waals heterostructure(HS) on which all the measurements were performed. The boundary of the CrI_3 and WSe_2 overlap region has been marked with yellow dashed line. We found 0D X^+ emissions at nano-pillar locations identified as P1-P5 in white dashed circles.

2 Magnetic circular dichroism(MCD) in bilayer CrI_3

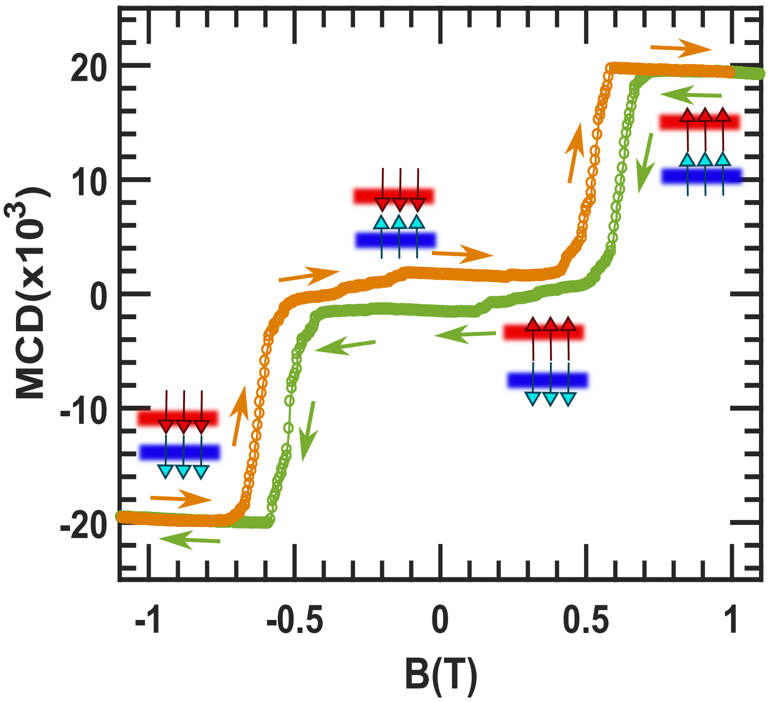

Supplementary Figure 2. MCD in bilayer CrI_3

We have performed MCD measurements on bilayer CrI_3 . Spin-flip transitions cause a step- like change in MCD signal as a function of applied magnetic field. The fields at which such changes occur agree well in the MCD and PL polarization(ρ) measurements shown in Fig. 2(c) of the main manuscript.

3 Measured photo-luminescence spectra for other 0D X^+

In this figure we have shown the PL spectra from the 5 nano-pillar sites that were identified in Supplementary Figure 1. In Supplementary Figure 3(a) The PL spectra at no applied magnetic field is shown. We can see multiple 0D X^0 as well as 0D X^+ at every pillar site. The presence of multiple sharp emission lines is a tell-tale signature of strain due to nano-pillars. Supplementary Figure 3(b) shows magneto-PL spectra at pillar site 5. We clearly see characteristic X shaped dispersion for the emitters marked 0D X^+ . In the main manuscript we have studied one 0D X^+ from pillar 4. In contrast, the emitters marked 0D X^0 show a more quadratic dispersion at low fields and a finite zero field splitting because of un-quenched electron and hole exchange interactions.

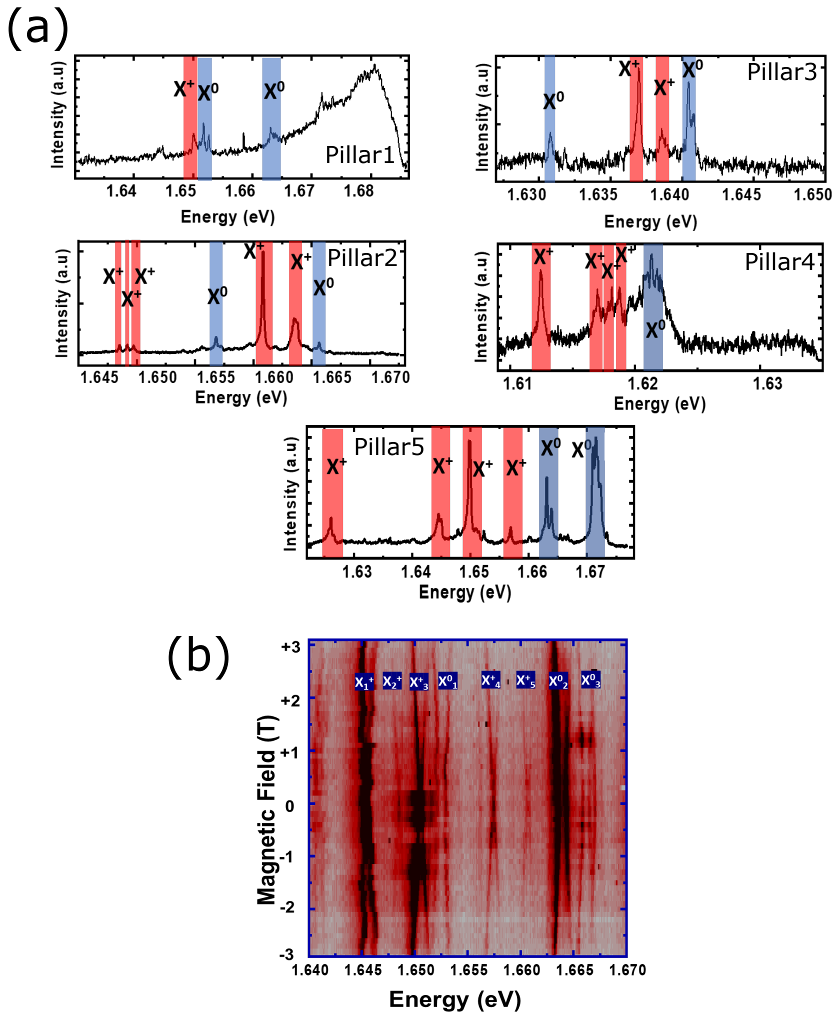

Supplementary Figure 3 (a) PL spectra at zero applied field for five different nanopillar sites

(b) An exemplary magneto-PL spectra taken at Pillar 5

4 FSS of 0D X^0

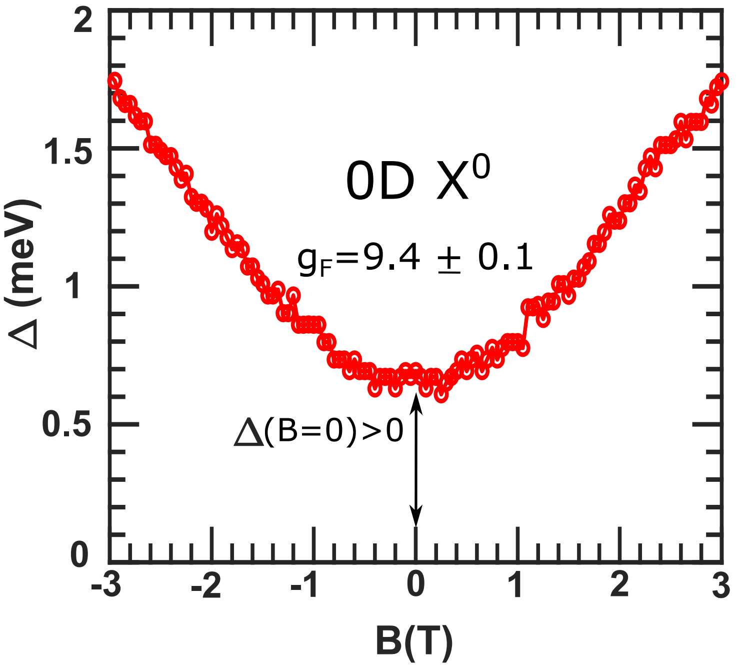

Supplementary Figure 4. Fine Structure Splitting of 0D X^0 as a function of B

To differentiate between magneto-optical behaviors of 0D X^+ and 0D X^0, we also performed PL spectroscopy on 0D X^0 at various B fields in the Faraday configuration. As clearly shown on recent studies,the existence of the anisotropic electron–hole exchange interaction in 0D X^0 leads to a doublet spectra at zero magnetic field, respectively. Supplementary Figure 4 displays a typical PL intensity map as function of applied B fields for 0D X^0 which is trapped in the shallow confinement potential induced by localized strain pockets formed by pillars. As expected, a zero field FSS of approximately 800 μeV is clearly observable in 0D X^0, which is consistent with previously reported a zero field FSS ranged between 500 μeV and 1000 μeV. In addition, 0D X^0 shows a quadratic dispersion behavior for small fields, while 0D X^+ as mentioned earlier exhibits

X shape dispersion behavior. In 0D X^0, the FSS increases as function of applied magnetic field by $\Delta E = \sqrt{(\Delta_0)^2 + (g_F \mu_B B)^2}$, wherein Δ_0 is a zero-field FSS. We find that this 0D X^0 has the Landé g-factor of 9.4 ± 0.1, which is comparable to previous reports for localized 0D X^+ Landé g-factors in WSe_2

5 Pump power dependent intensity of 0D X^+

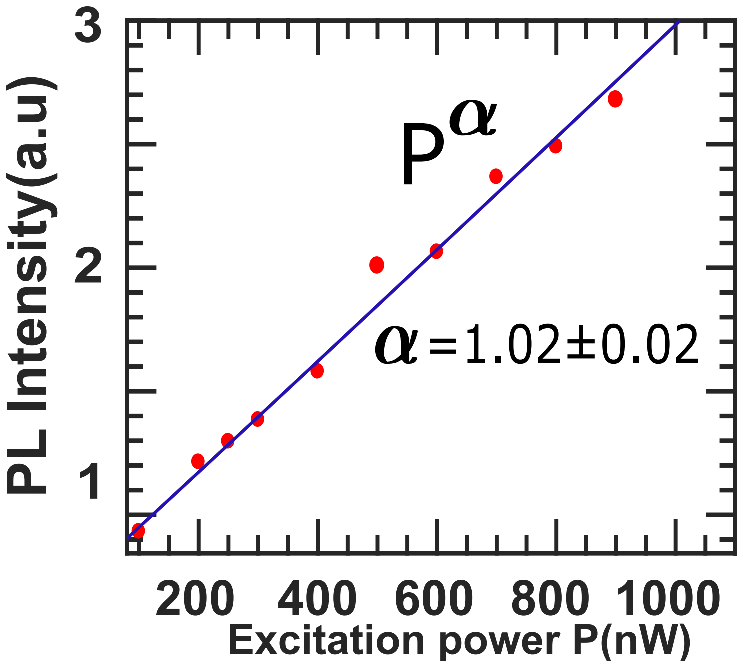

Supplementary Figure 5. Pump power dependent intensity of 0D X^+

We have performed pump-power dependent measurements on the intensity of 0D X^+ . We found that the increase in intensity is close to linear, thereby ruling out the possibility of charged bi-excitonic species.
